# The spatial parameters of gait and their association with falls, functional decline and death in older adults: a prospective study

**DOI:** 10.1038/s41598-019-45113-2

**Published:** 2019-06-19

**Authors:** Alejandro Rodríguez-Molinero, Alexandra Herrero-Larrea, Antonio Miñarro, Leire Narvaiza, César Gálvez-Barrón, Natalia Gonzalo León, Esther Valldosera, Eva de Mingo, Oscar Macho, David Aivar, Efren Pinzón, Adilis Alba, Jorge Passarelli, Nadia Stasi, Rosa Ana Valverde, Liane Kruse, Elisabet Felipe, Isabel Collado, Joan Bosch Sabater

**Affiliations:** 1 Àrea de recerca, Consorci Sanitari de l’Alt Penedès i Garraf, Vilafranca del Penedès, Spain; 2 Departament de Genètica, Microbiologia i Estadística, Facultat de Biología, Universitat de Barcelona, Barcelona, Spain; 3 Unitat de Psicogeriatría, Hospital Benito Menni, Sant Boi de Llobregat, Spain; 4 Fundació Privada Sant Antoni Abat, Vilanova i la Geltrú, Spain; 5 Universitat Internacional de Catalunya. Sant Cugat del Vallès, Barcelona, Spain

**Keywords:** Risk factors, Disability

## Abstract

Association between spatial gait parameters and adverse health outcomes in the elderly has not been sufficiently studied. The goal of this study is to evaluate whether the stride length or the step width predict falls, functional loss and mortality. We conducted a prospective cohort study on a probabilistic sample of 431 noninstitutionalized, older-than-64-years subjects living in Spain, who were followed-up for five years. In the baseline visit, spatial gait parameters were recorded along with several control variables, with special emphasis on known medical conditions, strength, balance and functional and cognitive capacities. In the follow-up calls, vital status, functional status and number of falls from last control were recorded. We found that a normalized-to-height stride length shorter than 0.52 predicted recurrent falls in the next 6 months with 93% sensitivity and 53% specificity (AUC: 0.72), and in the next 12 months with 81% sensitivity and 57% specificity (AUC: 0.67). A normalized stride length <0.5 predicted functional loss at 12 months with a sensitivity of 79.4% and specificity of 65.6% (AUC: 0.75). This predictive capacity remained independent after correcting for the rest of risk factors studied. Step-with was not clearly related to functional loss or falls. Both shorter normalized stride length (OR1.56; AUC: 0.62; p < 0.05) and larger step width (OR1.42; AUC: 0.62; p < 0.05) were associated with risk of death at 60 months; however, none of them remained as independent predictor of death, after correcting for other risk factors. In summary, spatial gait parameters may be risk markers for adverse outcomes in the elderly. Step length is independently associated with functional loss and falls at one year, after correction for numerous known risk factors.

## Introduction

Gait abnormalities can cause falls and decrease of mobility, with important repercussions for the health. 82% of the people older than 85 years old present gait abnormalities, in many cases due to osteoarticular or neuromuscular pathologies, which are easily recognizable by clinicians^1–3^. In other cases, however, alterations in the gait of the elderly are presented isolated, without an obvious pathology that causes them^2,4^. This type of gait abnormalities may correspond to preclinical states of cardiovascular and neurodegenerative diseases, as suggested by the fact that they are associated with mortality and represent a risk marker of developing these pathologies in a future time^5–8^.

The reduction in gait speed is frequently used as a marker of frailty, since it has been related to functional loss and death, although its relationship with falls has been inconsistent throughout several studies^9–11^. There are very few prospective studies examining the association of other gait parameters with adverse health outcomes in the elderly. In particular, the spatial gait parameters (stride length and step width) have been very little studied.

The stride length (or the step length; one stride is 2 steps) is an extremely easy parameter to measure in clinical practice, as it can be calculated by counting the number of steps necessary to cover a known distance. Despite the simplicity of this measure, very few prospective studies have analyzed its predictive capacity for adverse health outcomes in the elderly^12–18^. The stride length is related to gait speed, but this does not means that it has the same predictive capacity for health events, as has been shown occasionally, in the few prospective studies that analyzed both^17^.

The step width it is not so easy to quantify, since technological means are usually required for an accurate measurement. However, it may also be of interest to measure its predictive capacity for adverse health outcomes, especially in the case of falls, as some fall scales use it as risk marker (ex. the Tinetti scale^19^), without the association having been confirmed in other prospective studies.

In this study we analyze the predictive capacity of the spatial parameters of the gait (width and length of the stride), for some adverse health events of the elderly: falls, functional loss and death. Importantly, all gait parameters where recorded indoors at elderly people homes. This is an unusual feature of our database that offers a more-realistic picture of the elderly people gait, and thus will potentially serve better as reference standard when the new technologies available today, evolve to the point of reliably measuring the gait on an outpatient basis^20^.

## Methods

This longitudinal, prospective study of predictive validity involved a cohort of non-institutionalized, older-than-64-years subjects living in Spain. Participants unable to walk autonomously (without the aid of a third person) or needing walking aids other than canes were excluded. The study included a face-to-face baseline visit and telephone follow-up visits for five years (at months 4, 6, 9, 12 and 60). Additional mortality data, corresponding to the studied period, were obtained from the Spanish National Statistics Institute. The fieldwork was conducted between years 2007 and 2015.

Protocol for collecting the data of baseline visit, along with sampling and quality control procedures, have been previously published in the journal^20^.

In brief, in the baseline visit, sociodemographic data were recorded (age, sex, education level, cohabitation status), as well as the list of medicines used by every participant and a list of previous diseases, according to the questionnaire of the Spanish National Health Survey 2006^21^. Cognitive capacity was measured with the Pfeiffer’s test^22^ and depression was screened by using the GDS-5^23^. Previous falls in a 6 month period were also recorded. All the participants were weighed and their height was measured, muscular strength was assessed by using the Medical Research Council Scale^24^ and balance was assessed by using the first 4 items of the Tinetti balance scale (balance in a sitting position, the ability to stand without assistance, balance in standing position and immediate standing)^19^. Functional capacity was measured with the Katz index^25^, which measures the degree of dependence for the following basic activities of daily living (BADL): bathing, dressing, toileting, transferring, continence, and feeding (scores from 0 to 6: 0 for total independence and 6 dependence for all activities).

Spatial gait parameters were recorded at the participants’ homes using an ink footprint record, so that the prints could be preserved, transported and later analyzed^26–28^. The footprint method consist in making the participants walk on a paper spread on the floor, with the soles of the shoes impregnated with ink. The detailed method for obtaining printed footprints can also be found in the previous publication of the baseline data^20^.

The process of recording of gait parameters was videotaped and all the videos were reviewed by two physiotherapists with experience in gait analysis. All the tests that, at the discretion of any of the two physiotherapists, contained artifacts or lack of naturalness in walking were excluded from the study. Most of the exclusions corresponded to participants who paid attention to the gait recording method (ie. looking to their feet while walking), or to those who experienced adherence to the paper in the first steps. Adherence can happen when the participants stand on the paper for a long time, after their soles have been impregnated with ink. In the protocol there were measures planned to avoid this circumstance, but the protocol was not well followed in all cases at this point.

In the follow-up telephone calls, we recorded the Katz index for basic activities of daily living, the participants’ vital status and cause of death, and the number of falls experienced by the subject from the baseline visit or the previous telephone call (a fall was defined as any event, where the subject unintentionally came to rest on the ground or a lower level) Also, whether the patient had started rehabilitation programs and had been hospitalized or institutionalized from the last contact call was recorded. The people in charge of the follow-up used a structured interview model. They had received theoretical and practical training in the administration of the interviews, and were blind to the participant’s gait parameters data.

The protocol was conducted in accordance with the principles of Helsinki Delaration and approved by the Consoric Sanitari del Mresme Ethical Committee. All participants signed informed consent before inclusion.

### Statistical analysis

Sample size was calculated for falls and functional loss objectives. A size of 60 individuals per group was estimated to be sufficient for finding a difference between fallers and non-fallers of 0.08 in normalized stride length (pilot variance 0.02) or 2.5 cm in step width (pilot variance 19 cm), considering a design effect of 1.5, an alpha error 0.05 and a 80% power. Also, considering the functional loss as a continuous response variable, a sample size of 302 subjects was calculated to be sufficient to estimate functional loss at one year, with a 0.05 alpha error and a 90% power and including a design effect of 1.5. For such calculation the global significance test for the regression was used (*f* = *R²*/1 − *R*²) assuming an effect size value (*f*) of 0.053 and *R²* ~ 0:05^29,30^.

The sample was weighted according to the population in the 2012 annual report of the Spanish National Institute of Statistics (INE).

Stride length and step width were recorded and analyzed in centimeters, as quantitative continuous variables. Stride length was normalized to the subject’s height and the step width was related to the stride length using the following formula^20^:

$${\rm{ratio}}\,{\rm{width}}\,{\rm{to}}\,{\rm{length}}={\rm{step}}\,{\rm{with}}/{\rm{normalized}}\,{\rm{stride}}\,{\rm{length}}$$

Functional loss at any given follow-up period was defined as the difference between the Katz’s index at that follow-up point and the baseline Katz’s index. Incident disability was considered when Katz’s index rose from 0 to any other score, during the follow-up period. For the functional outcomes, we excluded the follow up period subsequent to the occurrence of events that can abruptly change the functional performance: hospitalization, institutionalization or rehabilitation programs. When analyzing associations between gait parameters and incident falls, we also excluded data from follow-up periods after any of these two events: 1.- Changes in medicines that confer fall risk (benzodiazepines, antidepressants, neuroleptics, digoxin and antiarrhythmics A1c); 2.-start of physiotherapy or occupational therapy interventions aimed at reducing the risk of falls. The reason for these exclusions is that these iatrogenic changes can modify the risk of falls after the gait parameters were recorded in the baseline and therefore bias the results.

Difference in means of the gait parameters were analyzed between individuals with repeated falls (2 or more) and the rest of the sample, during the first six and twelve months of follow-up. Analysis was stratified for sex, and the joint effect of sex and fall-risk on gait parameters was studied by using ANOVA. Association between gait parameters and incident falls was studied by using the Poisson regression. In the case of gait parameters markedly associated with incident falls, we also studied the validity (sensitivity, specificity and AUC) of different cut points of the parameter, for recurrent falls prediction.

Association between gait parameters and functional status at baseline (basal Katz index) was studied by using the Poisson regression, and their association with functional loss during follow-up (change from Katz at baseline) was analyzed by using linear regression models. Also, differences in means of the gait parameters were analyzed between individuals with and without functional loss at one year. Although functional status was collected in all follow-up calls up one year, we only present in the paper the results of the functional outcomes at 1 year (intermediate follow-up data are not shown).

Mortality was treated as a dichotomous variable, and its association with baseline gait parameters was studied by using logistic regression models, and by analyzing the difference in means of the gait parameters, between deceased and alive participants. The analysis of mortality at five years is presented, since it is the period with the greatest number of events which allows drawing more significant results.

To confirm the different associations found in the bivariate analysis, multivariable models were built for falls (Poisson regression), functional outcomes (linear regression models) and mortality (logistic regression models) including the other relevant risk factors recorded at baseline.

Data were analyzed by using the statistical package R v. 3.3.1. The datasets generated and analyzed during the current study are available from the corresponding author on reasonable request.

## Results

### Sample Characteristics

**Figure 1:**
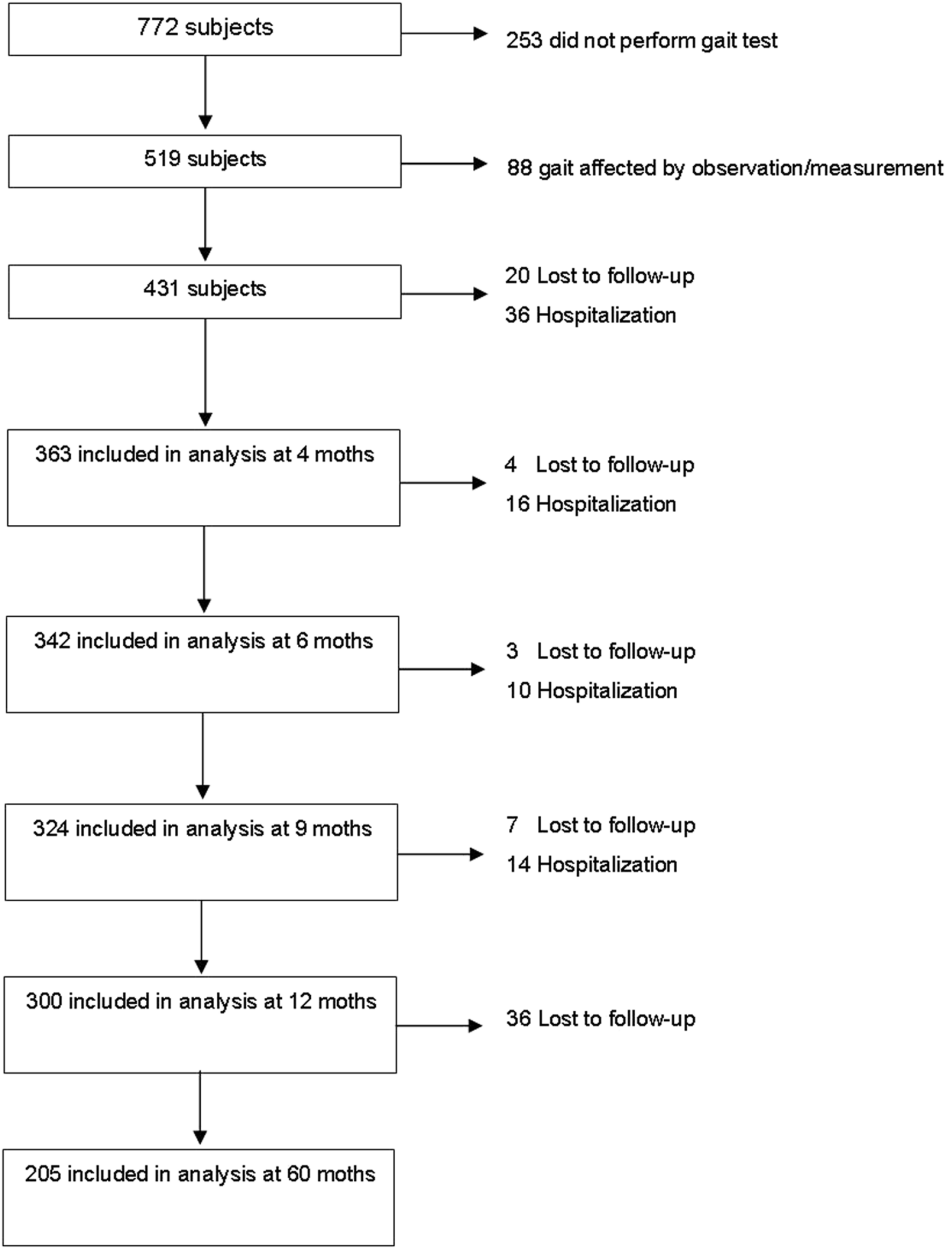
Sample flow chart.

Of the 772 participants initially included, 519 were able to walk without assistance and completed the footprints test. Of them, 88 were excluded from the analysis, due to doubts on the “naturalness” of the gait, by any of the two experts who reviewed the videos of the test, thus 431 subjects were finally included. Details of recruitment and inclusion of patients are shown in Fig. 1. Baseline characteristics of the sample were reported previously^20^. The baseline characteristics participants with complete data and at least one telephone control are shown in Table 1.

**Table 1:** Socio-demographic and health data of participants who completed the first follow-up period.

	Mean	SD
**Age** (years)	75.8	7
**Height** (cm)	159.5	8.9
**IMC** (kg/m^2^)	28.9	4.6
**Stride length** (cm)	89.3	25.8
**Step width** (cm)	10.2	4.1
	**Median**	**IQR**
**Comorbidity** ^a^	3	3
**Polypharmacy** ^b^	4	4
	**n**	**%**
**Sex**		
Male	158	42.7
Female	211	57.3
**Education level**		
None	92	25
Basic	207	56.4
Intermediate	39	10.6
University	29	7.9
**Cohabitation**		
Lives alone	96	26.3
Cohabitates with someone	269	73.7
**Katz**		
0	293	79.3
01-Feb	71	19.1
03-Apr	2	0.6
05-Jun	4	1
**Pfeiffer**		
0–3	349	94.8
04-Jul	17	4.6
08-Oct	3	0.6

^a^Number of chronic conditions.

^b^Number of chronic medications used.

### Spatial gait parameters and falls

An average of 4 strides were analyzed per person (two from each side).The group of participants with multiple falls at year one, had an average shorter stride length in the baseline: 74.0 cm vs 91.4 cm (difference in means: −17.462; CI95%: −32.500, −2.424; p < 0.05). Baseline step width was not found to be different between the elderly participants who fell repeatedly, and those with no falls or a single fall. Mean differences in all spatial gait parameters, in the whole sample and within sex groups, are shown in Table 2. The ratio width to normalized length was different between recurrent fallers and the rest of the sample (p < 0.001), but not between sex groups. Bivariated regression analysis and odds ratio of each gait parameter for recurrent falls, and accumulated falls is depicted in the Table 3.

**Table 2:** Mean differences in all spatial gait parameters.

		MONTH 6			MONTH 12		
		Fallers*	No Fallers	p	Fallers*	No Fallers	p
Stride length	Total	66,2	90,9	<**0**,**001**	74,0	91,4	**0**,**023**
	Male	67,6	103,4	**0**,**001**	68,2	104,1	<**0**,**001**
	Female	65,9	81,3	**0**,**006**	73,4	81,3	0,519
		**Fallers**	**No Fallers**	**p**	**Fallers**	**No Fallers**	**p**
Normalized stride length	Total	0,43	0,57	<**0**,**001**	0,48	0,57	0,058
	Male	0,39	0,62	<**0**,**001**	0,40	0,63	<**0**,**001**
	Female	0,44	0,53	**0**,**009**	0,5	0,53	0,602
		**Fallers**	**No Fallers**	**p**	**Fallers**	**No Fallers**	**p**
Step width	Total	11,1	10,2	0,444	10,6	10,2	0,675
	Male	12,8	10,3	0,276	12,7	10,3	0,230
	Female	10,7	10,0	0,650	10,1	10,0	0,951
		**Fallers**	**No Fallers**	**p**	**Fallers**	**No Fallers**	**p**
**Ratio width to normalized length**	Total	27,7	20,8	**0**,**042**	25,4	20,3	0,200
	Male	29,1	19,0	<**0**,**001**	29,0	18,5	<**0**,**001**
	Female	27,5	22,2	0,275	24,5	21,7	0,700

*Faller: 2 or more falls within the studied period.

**Table 3:** Bivariated regression analysis and odds ratio of each gait parameter for recurrent falls, and accumulated falls.

	Variable	Estimator	p	OR	IC95%	
Accumulated falls (6 months)	Normalized stride length	−0,43	<**0**,**001**	0,65	0,53	0,79
	Step width	0,10	0,311	1,10	0,92	1,32
	Ratio width to normalized length	0,03	<**0**,**001**	1,03	1,00	1,06
Accumulated falls (12 months)	Normalized stride length	−4,57	<**0**,**001**	0,63	0,51	0,79
	Step width	0,09	0,31	1,10	0,92	1,31
	Ratio width to normalized length	0,03	<**0**,**001**	1,03	1,00	1,06
Recurrent falls* (6 months)	Normalized stride length	−0,61	<**0**,**001**	0,54	0,41	0,71
	Step width	0,05	0,419	1,05	0,93	1,19
	Ratio width to normalized length	0,03	**0**,**029**	1,03	1,00	1,05
Recurrent falls* (12 months)	Normalized stride length	−0,45	0,076	0,64	0,39	1,05
	Step width	0,03	0,666	1,03	0,91	1,16
	Ratio width to normalized length	0,02	0,092	1,02	1,00	1,05

^*^Recurrent falls: two or more falls in a given period.

Bold type: significant finding.

A normalized stride length shorter than 0.52 predicted recurrent falls in the next 6 months with 93% sensitivity and 53% specificity (AUC: 0.72; CI95%: 0.61–0.82), and recurrent falls in the next 12 months with 81% sensitivity and 57% specificity (AUC: 0.67; CI95%: 0.56–0.79). Table 4 shows the probability of recurrent falls for different values of normalized stride length.

**Table 4:** Risk of falls, functional decline and dependency associated to different normalized stride length values.

Normalized stride length	% of fallers^a^		% functional loss^b^	% incident disability^c^
	Month 6	Month 12	Month 12	Month 12
0,16	29,5	26,9	44,9	67,8
0,25	19,6	19,9	32,5	59,2
0,33	12,5	14,4	22,2	50
0,42	7,7	10,2	14,5	40,9
0,51	4,6	7,1	9,1	32,3
0,60	2,7	4,9	5,6	24,7
0,69	1,6	3,4	3,4	18,5
0,77	1,0	2,3	2	13,5
0,86	0,6	1,6	1,2	9,7
0,95	0,3	1,1	0,7	6,9

^a^Faller: two or more falls in the studied period.

^b^Functional loss: change in Katz index from baseline.

^c^Incident disability: new dependency from any ABVD (any increment in Katz index from 0).

The following control variables were associated with falls in the subsequent year: age (p < 0.01), sex (p < 0.05), impaired balance (p < 0.001), depression (P < 0.001) and polypharmacy (p < 0.05). Multi-variable models were built including gait parameters and other relevant baseline variables. Both short normalized stride length and large ratio width to length, resulted to be independent predictors for falls that remained in the final models (Table 5).

**Table 5:** Final multivariate adjusted models for recurrent falls prediction.

	Estimator	p	exp(coefic)	IC95%	
**Multivariate model for the normalized stride length**					
Normalized stride length	−8,65	**0**,**001**	0,00	0,00	0,03
Strength	0,23	**0**,**014**	1,26	1,05	1,50
Cognition (Pfeiffer)	0,99	**<0**,**001**	2,68	1,78	4,04
Depression (GDS5)	−1,17	**0**,**007**	0,31	0,14	0,72
Comorbidity	−0,73	**0**,**022**	0,48	0,26	0,89
Polypharmacy	0,50	**0**,**002**	1,65	1,21	2,24
**Multivariate model for the ratio width to length**					
Ratio width to length	0.93	**0**,**011**	2,52	1,26	5,05
Cognition (Pfeiffer)	1,04	**<0**,**001**	2,82	1,75	4,53
Depression (GDS5)	−0.72	**0**,**046**	0,49	0,24	0,98
Comorbidity	−0,71	**0**,**023**	0,50	0,27	0,90
Polypharmacy	0,42	**0**,**003**	1,52	1,16	1,98

Bold type: significant finding.

### Spatial gait parameters and functional loss

Participants with disability at baseline (Katz > 0) had a shorter stride length: 80.1 cm vs 90.7 cm (difference in means: −10.6 cm; IC95%: −3.2 to −18.1; p < 0.01); and a shorter stride length in proportion to their height (normalized stride length): 0.51 vs 0.57 (difference in means: −0.06; CI95%: −0.02 to −0.10; p < 0.01). Also, participants with incident disability during the first year of follow-up (any increment from Katz = 0) had a shorter stride length: 82.3 cm vs 96.7 cm (difference in means:−14.4 cm; CI95%: −6.6 to −22.1; p < 0.001); and shorter normalized stride length: 0.52 vs 0.60 (difference in means: −0.08; CI95%: −0.04 to −0.13; p = 0.001). A normalized stride length less than 0.5 (stride less than half the height) predicted functional loss at 12 months (worsening of the Katz index) with a sensitivity of 79.4%, specificity of 65.6% and area under the ROC curve of 0.75. Functional loss Odds Ratio was 1.19 (CI95%: 1.10–1.29) for each decrement of 0.1 units in normalized stride length. Table 4 shows the probability of functional loss and incident disability for different values of normalized stride length.

No significant differences were found in the step width between participants without disability and those with disability at baseline or who developed it throughout the follow-up. However, the ratio between the step width and the normalized stride length, was associated with incident disability at 12 months: 23.7 vs 18.1 (difference in means: 5.6, 95%; CI: 9.8 to 1.4; p < 0.01), meaning that people with shorter and wider steps are more likely to develop disability.

The following basal variables were associated with functional loss at 12 months: age (p < 0.05), sex (p < 0.001), impaired balance (p < 0.05), baseline functional capacity (p < 0.01), depression (P < 0.01), polypharmacy (p < 0.001) (Table 6). Multivariate models were built, in which gait parameters were included in combination with baseline risk factors for functional loss. The predictive capacity of the normalized stride length for functional loss at one year was independent of the other risk factors studied (Table 6); however, the ratio between the width and the normalized stride length, lost its predictive capacity on functional loss when the other risk factors were introduced in the model.

**Table 6:** Bivariate and multivariate analysis on the predictive capacity of normalized stride length and other basal variables on functional loss.

	Bivariate Analysis				Final Adjusted Model		
	Estimator	IC95%	T statistic	P	Estimator	IC95%	P
Normalized stride length	−1.72	−2.50; −0.94	—	<0.001	−1.27	−2.01; −0.54	0.001
Sex	−0.21	−0.40; −0.02	−2.19	0.030	—	—	—
Age	0.04	0.03; 0.05	5.35	<0.001	0.03	0.02; 0.05	<0.001
Body Mass Index	0.01	0.02; 0.03	0.54	0.588	—	—	—
Strength	−0.03	0.07; 0.01	−1.60	0.110	—	—	—
Balance	−0.13	−0.24; −0.01	−2.15	0.033	—	—	—
Functional ability (Katz)	−0.18	−0.31; −0.07	−2.66	0.008	−0.35	−0.50; −0.20	<0.001
Cognition (Pfeiffer)	0.08	−0.01; 0.17	1.70	0.091	—	—	—
Depression (GDS5)	0.13	0.04; 0.22	2.77	0.006	—	—	—
Comorbidity	0.02	−0.05; 0.08	0.50	0.620	—	—	—
Polypharmacy	0.08	0.03; 0.12	3.50	0.001	0.04	0.00; 0.08	0.046
Previous falls	0.19	−0.06; 0.44	1.48	0.141	—	—	—

### Spatial gait parameters and mortality at five years

The global mortality rate at 60 months (5 years) was 22.3% (IC95%: 18%-27.8%). Bivariated regression analysis showed that all the studied spatial gait parameters were associated with increased risk of death at 60 months: shorter normalized stride length (OR 1.56; AUC: 0.62; p < 0.05), larger step width (OR 1.42.; AUC: 0.62; p < 0.05) and larger step width in proportion to the normalized stride length (OR 1.78; AUC: 0.64; p < 0.001). Means analysis showed that the mean of step width in proportion to the normalized stride length was significantly different between dead and survivors: 30.0 vs 20.2 (difference in means: 9.9; CI95%: 17.0 to 2.7; p < 0.01), not being significant the difference in means of the two parameters separately.

Of the baseline variables studied, the following were associated with mortality at 5 years: age (p < 0.001), muscle strength (p < 0.01), impaired balance (p < 0.01), baseline functional capacity (p < 0.01), baseline cognitive function (p < 0.01) and depression (p < 0.05). Multivariate models were built, in which, in addition to these variables, BMI, diabetes, COPD, heart disease, comorbidity, polypharmacy and previous falls were introduced. It was observed that none of the gait parameters studied predicted mortality at 5 years independently of the other risk factors introduced in the models.

## Discussion

### Main observations

This prospective study of the normal gait at home showed that the normalized stride length is predictive of falls and functional loss in older adults. The normalized stride length kept its predictive capacity even after correcting for a large number of factors associated with falls and functional loss, including the baseline functional capacity of the subject. A stride length shorter than half of the height is a sensitive risk marker for functional loss and falls. Step width was not independently associated to these outcomes, though the proportion of the step width to the normalized stride length showed some predictive properties for incident falls. All gait parameters are also associated with mortality at 5 years, though this association was not independent from other risk factors of death in the elderly.

### Stride length

Although several case-control studies and some prospective studies in specific populations have assessed the association between falls and step length or stride length (the stride length is twice the step length), we have found a single prospective study in which, like us, the authors measured normal gait at normal pace in general elderly population. In this study, Verghese *et al*. measured gait in 597 adults aged 70 and older, using the GaitRite walkway, and found that stride length was a weak predictor for falls, after adjusting by sociodemographic variables (sex, age and education level)^16^. However, stride length lost its predictive capacity when other health related variables were introduced in their model. Conversely, we found on our data, a strong and independent association between the risk of fall and the stride length, after correcting for a large number of health variables. The most striking difference between both studies is that we conducted our study at the elderly participants’ home, minimizing the selection bias related to commuting to a gait laboratory for the study procedures; thus our sample may differ markedly from theirs, being possible that the elders of our sample had a worse walking ability than those included in theirs. As far as we know, no other authors have measured the spatial gait parameters at home.

Although the association between gait speed and disability and mortality in older people has been repeatedly demonstrated^11^, scarce research studies have addressed the stride length predictive capacity for the occurrence of adverse health events. In a prospective study, the stride length predicted disability to walk (walking half a mile) in a sample of aged people, although such a prediction did not seem to add information to gait speed, with which it was co-linear^18^ Up to our knowledge, only one prospective study addressed the relationship between stride length and disability for BADL and mortality. Woo *et al*. measured the stride length in a Chinese population sample by counting the steps subjects needed to cover an 18-feet distance (≈5.5 meters)^17^. In that study, they found that the stride length was predictive of mortality and disability for BADL at 36 months (measured with the Barthel’s index^31^), and importantly, the prediction was independent of gait speed.

According to our data, the normalized stride length is high sensitive for detecting subjects at risk of recurrent falling in the first 6 months (very few fallers has large steps), though its specificity could be better (half of non-fallers screened positive in the test). Also, about 80% of people with recurrent falls or functional loss in the next year, showed in the basal point a stride length less than half of their height. Therefore, the normalized stride length may be clinically useful as a first screening test or in combination with other risk markers for falls and functional loss. It should be taken into account that stride length is an easily measurable parameter in the clinical practice (though for research purposes we used a complex method here, stride length can be easily obtained by counting the steps given to walk a known distance). Finally, considering that we have found that normalized stride length is predictive with falls, mortality and dependence, it is possible that the normalized stride length is also a good marker of fragility; this possibility should be pursued in further studies including other frailty markers and more frailty syndrome-related health events.

### Step width

Step width is relatively hard to record and requires laborious techniques such as footprints methods (here used) or expensive systems such as computerized walkways, which are also impractical to study gait at home. Although this parameter is an indicator of future disability and mortality, it is not independent of parameters such as age or baseline functional situation, which are more strongly associated with the appearance of adverse health outcomes. Although some studies exist analyzing step width variability as a predictor of falls^32^, we only have found a previous prospective study on step width itself. In this study step width showed weak association with falls, but they did not adjust the analysis by a number of other relevant parameters^33^.

No other prospective studies studying the relationship between the step width and functional decline or mortality on general population were found. Therefore, although the associations we found were not independent of other risk factors, our results are novel and may provide new insight in the field.

As far as we know, no other authors have studied the relationship between step width and normalized stride length. This interesting parameter which merges normalized stride length and width, it is not affected by sex, it is independently associated to incident falls, and a risk marker of functional loss. However, it does not look a better predictor than normalized stride length alone, and it is much difficult to be measured, so it seems impractical its clinical application to us.

### Limitations

We did not measure gait speed, which has been clearly associated with functional decline, and also with falls, although with conflicting results. Therefore, we cannot establish direct comparisons with the normalized stride length associations found here. However, comparing with previous published results, it does not seem that the predictive capacity of the normalized stride length is inferior to that of the gait speed. Woo *et al*. did in fact a direct comparison between the predictive capacity of stride length and gait speed, being the first greater for disability and mortality^17^. Also, the predictive capacity of the gait speed for falls has been recently reported to be lower than believed, and lower than that found by us for the normalized stride length (AUC: 0.50 for falls and AUC: 0.59 for recurrent falls in one year; according to the authors, the results were not better at 6 months of follow-up)^34^.

Another limitation of this study was the reduction in the sample size, which was due to two main causes: on the one hand, not all the initially recruited elders could walk without the help of a third person; on the other hand, as a result of the quality-control of the gait test, part of the sample had to be excluded due to unnatural gait, as judged by any of two evaluators. Such a sample reduction may impair the generalization of results although, statistically, the final sample size was enough to evaluate the studied associations. Even so, the final sample size may be considered large, when compared with those of other published studies, where difficult-to-measure gait parameters, such as the step width, were evaluated.

Global Cognitive function was introduced in the adjusted analysis because it has been consistently associated with falls, functional decline and mortality^35–37^. However, it would have been of interest to use a cognitive test which includes frontal lobe function, as it has been more directly related with gait changes^38,39^.

Lastly, the quarterly telephone calls, used to follow-up the incidence of falls, were separated enough for memory bias to occur^40^. Such a bias consists in the fact that elders tend to forget some falls, especially inconsequential ones. However, in our opinion, such a bias was not very important in our study, given that the global incidence of falls in our cohort (published elsewhere^41^) was very similar to those reported in many other studies of falls, some of them using more thorough recording methods.

## Conclusions

In conclusion, normalized stride length of the elderly, which is easy to measure in the clinical practice, shows a strong association with functional decline and falls, which is not accounted for by other known risk factors for these outcomes. No independent relationship has been observed between the absolute value of the step width and adverse health outcomes studied, though the proportion of the step width to the normalized stride length is related to the risk of falling.
